# Hormonal Balance, Photosynthesis, and Redox Reactions in the Leaves of *Caragana korshinskii* Kom. under Water Deficit

**DOI:** 10.3390/plants12112076

**Published:** 2023-05-23

**Authors:** Hui Yan, Xiaoli Liu, Hao Ding, Zhiguang Dai, Xiaoli Niu, Long Zhao

**Affiliations:** 1 College of Agricultural Equipment Engineering, Henan University of Science and Technology, Luoyang 471000, China; 2 Science and Technology Development Office, Henan University of Science and Technology, Luoyang 471000, China

**Keywords:** *Caragana*, different irrigation strategies, hormonal balance, photosynthesis, redox reaction

## Abstract

To evaluate the physiological responses of Korshinsk peashrub (*Caragana korshinskii* Kom.) to water deficit, photosynthetic gas exchange, chlorophyll fluorescence, and the levels of superoxide anion (O_2_^•−^), hydrogen peroxide (H_2_O_2_), malondialdehyde (MDA), antioxidant enzymes, and endogenous hormones in its leaves were investigated under different irrigation strategies during the entire growth period. The results showed that leaf growth-promoting hormones were maintained at a higher level during the stages of leaf expansion and vigorous growth, and zeatin riboside (ZR) and gibberellic acid (GA) gradually decreased with an increase in water deficit. At the leaf-shedding stage, the concentration of abscisic acid (ABA) dramatically increased, and the ratio of ABA to growth-promoting hormones increased to a high level, which indicated that the rate of leaf senescence and shedding was accelerated. At the stages of leaf expansion and vigorous growth, the actual efficiency of photosystem II (PSII) (Φ_PSii_) was downregulated with an increment in non-photochemical quenching (*NPQ*) under moderate water deficit. Excess excitation energy was dissipated, and the maximal efficiency of PSII (*F*_v_/*F*_m_) was maintained. However, with progressive water stress, the photo-protective mechanism was inadequate to avoid photo-damage; *F*_v_/*F*_m_ was decreased and photosynthesis was subject to non-stomatal inhibition under severe water deficit. At the leaf-shedding stage, non-stomatal factors became the major factors in limiting photosynthesis under moderate and severe water deficits. In addition, the generation of O_2_^•−^ and H_2_O_2_ in the leaves of *Caragana* was accelerated under moderate and severe water deficits, which caused an enhancement of antioxidant enzyme activities to maintain the oxidation–reduction balance. However, when the protective enzymes were insufficient in eliminating excessive reactive oxygen species (ROS), the activity of catalase (CAT) was reduced at the leaf-shedding stage. Taken all together, *Caragana* has strong drought resistance at the leaf expansion and vigorous growth stages, but weak drought resistance at the leaf-shedding stage.

## 1. Introduction

Korshinsk peashrub (*Caragana korshinskii* Kom.), a type of deciduous shrub, is mostly distributed in the semiarid and arid regions of northwestern China where it is primarily used to prevent desertification and raise livestock owing to its ecological and economic value [1,2]. Currently, water scarcity in these regions has become more serious with global warming, and the physiological properties of this shrub are impacted by associated water deficits. Substantial efforts have been made to reduce the adverse effects of water deficits on the growth of Korshinsk peashrub during recent decades. However, there has been little success because of inadequate knowledge regarding the physiological response mechanism of this plant to water scarcity. Therefore, the physiological responses of hormonal balance, photosynthesis, and redox reactions in the leaves of Korshinsk peashrub during an entire growth period were examined in our study.

Phytohormones, including abscisic acid (ABA), indole-3-acetic acid (IAA), zeatin riboside (ZR), and gibberellic acid (GA), are pivotal chemical signals that play an important role in regulating plant growth and development [3]. Water deficit can change endogenous hormone levels in crops, and various physiological responses, such as stomata movement, cell division, and leaf shedding, are, therefore, also affected. In general, the abovementioned phytohormones can be divided into two groups based on their functions. ABA is a growth-inhibiting hormone that can inhibit cell division and elongation and accelerate leaf senescence and shedding [4]. In contrast, IAA, ZR, and GA are growth-promoting hormones, which play important roles in promoting cell division and elongation and delaying leaf senescence [5]. Therefore, the relevant physiological processes are always regulated by hormonal interactions rather than just one hormone, and the effects of water deficit on endogenous hormones and hormonal balance merit further study.

Many current studies have reported that water deficit stimulates the variation of ABA and ZR in the roots, which are then transported by xylem sap to induce the closure of stomata [2,6]. Stomata are the primary passageways for the influx and outflux of CO_2_ in mesophyll cells, and their closure could restrict the availability of external CO_2_. When inadequate amounts of CO_2_ limit the demand of photosynthesis, stomatal factors can restrict the assimilation of photosynthetic carbon [7]. A reduction in the rate of photosynthesis caused by the closure of stomata could trigger an imbalance between the supplementation of photosynthetic electrons and the activity of the photochemical apparatus, which results in the over-excitation of absorbed light energy [8]. To avoid oxidative damage in the PSII reaction centers caused by over-reduction of the primary quinone electron acceptor, excess excitation energy is mostly dissipated as thermal energy [9]. However, when excess excitation energy cannot be effectively dissipated, the damage to photosynthetic apparatus and subsequent non-stomatal inhibition of photosynthetic carbon assimilation could affect physiological processes [2]. Thus, both stomatal and non-stomatal factors can participate in the restriction of photosynthesis. Currently, the factors that play a major role in limiting photosynthesis under water deficit stress are still debated. Some researchers have shown that stomatal closure is the dominant factor that restricts photosynthesis [10]. However, other studies have shown that non-stomatal factors are the primary reasons for a reduction in the rate of photosynthesis [11,12].

In addition, when there is an imbalance between the supply of electrons and the requirements for photosynthesis in plant physiological processes, excess electrons are transferred to singlet molecular oxygen. This results in the production of excessive amounts of reactive oxygen species (ROS), such as superoxide anion (O_2_^•−^) and hydrogen peroxide (H_2_O_2_). The generation of ROS results in oxidative damage to photosynthetic apparatus and a subsequent reduction in photosynthesis [13]. To avoid oxidative damage caused by the accumulation of ROS, a series of physiological mechanisms have been formed to effectively scavenge excess ROS. Superoxide dismutase (SOD), peroxidase (POD), and catalase (CAT) are the dominant antioxidant enzymes, and their activities have been reported to increase under water stress to scavenge excess ROS, maintaining the balance of cellular oxidoreduction and, thus, avoiding oxidative damage [14]. However, reductions in antioxidant enzyme activities and subsequent cellular oxidative damage could occur as the accumulation of ROS reaches a certain concentration [15].

To evaluate the physiological responses of Korshinsk peashrub to water scarcity, the hormonal balance, photosynthesis, and redox reactions in leaves subject to different irrigation strategies were studied during their entire growth period. The objective of this study was to investigate the following: (1) changes in the levels of endogenous hormones in the leaves during the entire growth period and the role of hormones in coordinating physiological responses under different irrigation strategies; (2) the primary factor that restricts the photosynthesis of Korshinsk peashrub during its entire growth period with different irrigation strategies and the mechanism of excitation energy utilization and dissipation at different growth stages; and (3) the accumulation of ROS and subsequent oxidative damage under water stress and the role of enzymatic antioxidants in alleviating oxidative damage. Our study provides theoretical guidance on how Korshinsk peashrub conducts deficit irrigation.

## 2. Materials and Methods

### 2.1. Plant Cultivation and Experimental Design

The research was conducted from April to October in 2021 at the Henan University of Science and Technology. One-year-old seedlings of *Caragana korshinskii* Kom. were planted in pots that were 22 cm in their lower diameter, 28 cm in their upper diameter, and 27 cm high. They were then filled with 14 kg of dry soil. Seedlings were irrigated to maintain 80% of field capacity before the experiments.

The water treatments were begun after the seedlings had germinated. The irrigation regimes included four different levels; i.e., 60–80% of field capacity (sufficient irrigation, SI), 50–70% of field capacity (mild water deficit, MiWD), 40–60% of field capacity (moderate water deficit, MoWD), and 30–50% of field capacity (severe water deficit, SWD). At leaf expansion, vigorous growth, and leaf-shedding stages, physiological parameters, including photosynthetic gas exchange, chlorophyll fluorescence, and the levels of O_2_^•−^, H_2_O_2_, malondialdehyde (MDA), antioxidant enzymes, and endogenous hormones in the leaves, were measured under different irrigation regimes. Twelve seedlings (three seedlings for each irrigation treatment) at each growth stage were selected for physiological analysis.

In addition, at the leaf-shedding stage, three seedlings of each irrigation regime were selected and divided into branches and leaves during their harvest, and the fresh weights of branches and leaves were immediately measured. Subsequently, the dry weights of branches and leaves were calculated from the fresh weight of these organs, and the fresh-to-dry mass ratio was determined by sampling.

### 2.2. Gas Exchange Measurements

Three mature leaves of each seedling were selected for gas exchange measurements. The photosynthetic rate (*P*_n_), stomatal conductance (*g*_s_), transpiration rate (Tr), intercellular CO_2_ concentration (*C*_i_), and ambient CO_2_ concentration (*C*_a_) were measured using a portable photosynthesis system (LI-6400; LI-COR Biosciences, Inc., Lincoln, NE, USA) as previously described [2]. The leaf stomatal limitation value (*L*_s_) was calculated as *L*_s_ = 1 − *C*_i_/*C*_a_ [16], and instantaneous water-use efficiency (_i_WUE) was calculated as _i_WUE = *P*_n_/T_r_ [17].

### 2.3. Measurements of Leaf Chlorophyll Fluorescence 

The leaves used for photosynthetic measurement were selected for chlorophyll fluorescence measurements. The maximal efficiency of photosystem II (PSII) (*F*_v_/*F*_m_), the actual efficiency of PSII (Ф_PSII_), and non-photochemical quenching (*NPQ*) were determined using a Mini-PAM chlorophyll fluorometer (Heinz Walz GmbH, Pfullingen, Germany) as previously described [18].

### 2.4. Determination of O_2_^•−^, H_2_O_2_, MDA, and the Activities of Antioxidant Enzymes 

The production rate of O_2_^•−^ and concentration of H_2_O_2_ in the leaves were spectrophotometrically assayed at 530 nm and 410 nm, respectively, using a TU-1810 spectrophotometer (Beijing Purkinje General Instrument Co., Ltd., Beijing, China) as described by He et al. [19].

Concentrations of MDA in the leaves were determined by spectrophotometry at 532, 600, and 450 nm, and calculated as follows: MDA (μmol g^−1^) = [6.45 × (A_532_ − A_600_) − 0.56 × A_450_] [20].

The activities of SOD, POD, and CAT in the leaves were spectrophotometrically assayed at 560 nm, 470 nm, and 240 nm, respectively, as previously described [20]. The soluble proteins in leaf tissues were also extracted and quantified as described by Luo et al. [21] to measure the activities of enzymes.

### 2.5. Determination of Phytohormones

The concentrations of ABA, IAA, GA, and ZR in the leaves were measured using an enzyme-linked immunosorbent assay (ELISA) as previously described [22].

### 2.6. Statistical Analysis

Statistical analyses were performed with SPSS 16.0 (SPSS, Inc., Chicago, IL, USA). Two-way analyses of variance (ANOVAs) were applied with the water treatments and growth period as two factors. The treatment means were compared for any significant differences at the 0.05 level with a Tukey’s HSD test.

## 3. Results

### 3.1. Growth Performance

**Figure 1:**
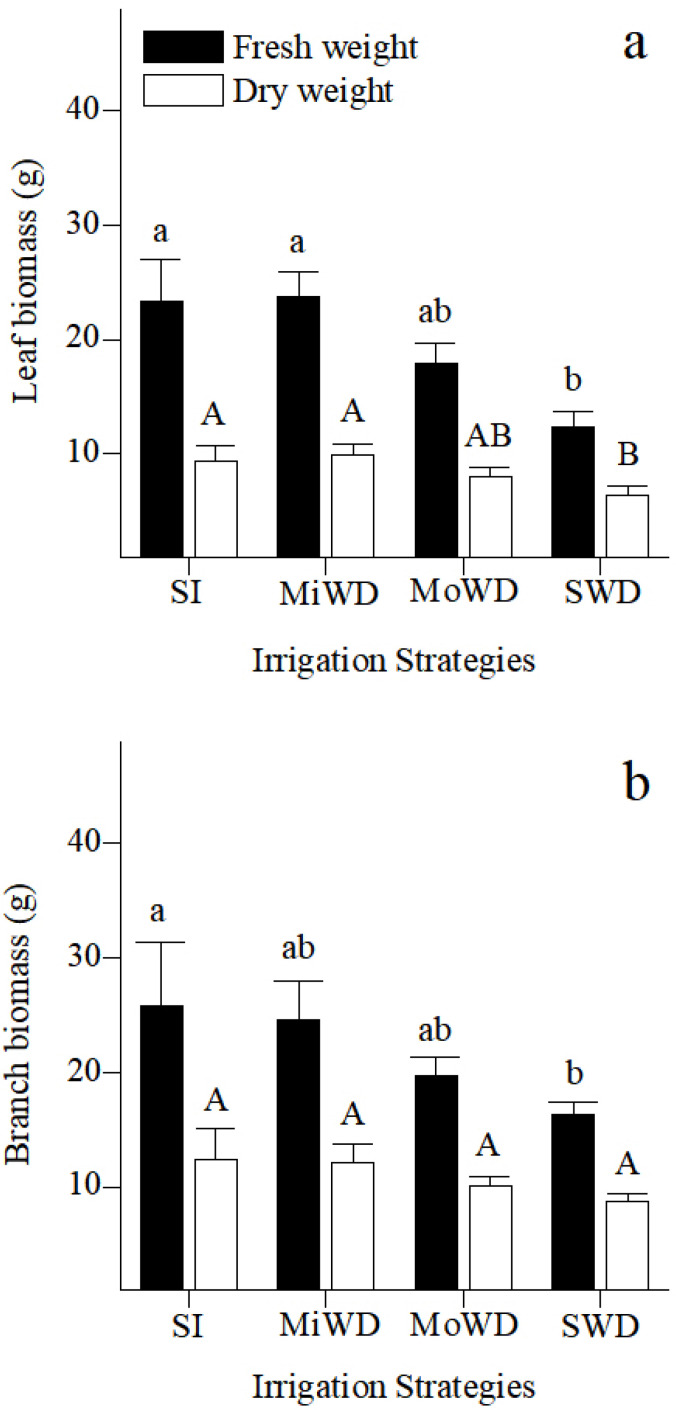
Changes in leaf biomass (**a**) and branch biomass (**b**) of *Caragana korshinskii* Kom. under different irrigation strategies. Data points are mean ± SD of three replicates. Different letters indicate significant difference among the treatments; (sufficient irrigation, SI), (mild water deficit, MiWD), (moderate water deficit, MoWD), and (severe water deficit, SWD).

The evolution of growth performance under different irrigation regimes is shown in Figure 1. With an increase in water deficit, the leaves’ fresh and dry weights tended to decrease, and the minimum values occurred under severe water deficit conditions. The variations in fresh weights of the branches were similar to those observed in the leaves, which gradually decreased with an increase in water deficit.

### 3.2. Endogenous Hormones and Their Ratios in Leaves

**Figure 2:**
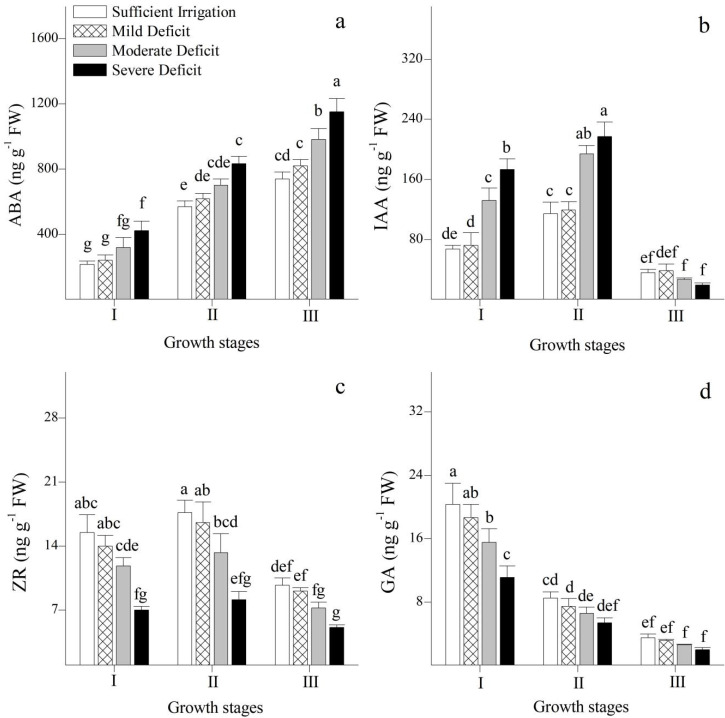
Changes in abscisic acid (ABA) (**a**), indole-3-acetic acid (IAA) (**b**), zeatin riboside (ZR) (**c**), and gibberellic acid (GA) (**d**) of *Caragana korshinskii* Kom. under different irrigation strategies at different growth stages. I, II, and III stand for leaf expansion stage, vigorous growth stage, and leaf-shedding stage, respectively. Data points are mean ± SD of three replicates. Different letters indicate significant difference among the treatments.

**Figure 3:**
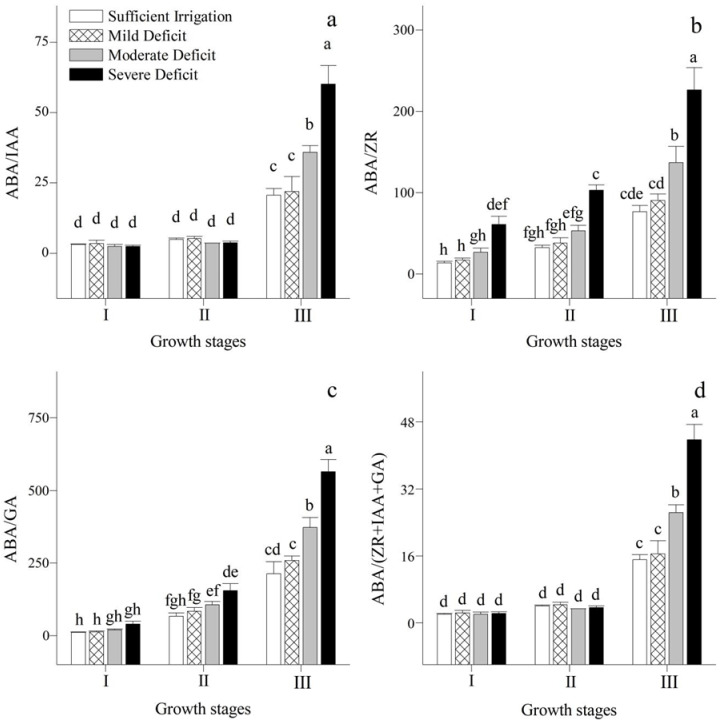
Changes in ABA/IAA (**a**), ABA/ZR (**b**), ABA/GA (**c**), and ABA/(ZR+IAA+GA) (**d**) of *Caragana korshinskii* Kom. under different irrigation strategies at different growth stages. I, II, and III stand for leaf expansion stage, vigorous growth stage, and leaf-shedding stage, respectively. Data points are mean ± SD of three replicates. Different letters indicate significant difference among the treatments.

The changes in ABA, IAA, ZR, GA, and their ratios in the leaves under different irrigation regimes were also analyzed in this study (Figure 2a–d and Figure 3a–d). The concentrations of IAA, ZR, and GA in the leaves were maintained at a higher level during the stages of leaf expansion and vigorous growth, and there were low ratios of ABA to IAA, ZR, GA, and (IAA + ZR + GA). However, when the leaves were shed, the concentration of ABA in the leaves was relatively high, and the ratios of ABA to other hormones rose. With an increment in levels of water deficit, the concentration of ABA and ratios of ABA/ZR and ABA/GA in the leaves increased, while those of ZR and GA gradually decreased. Under water deficit, the concentration of IAA increased at the stages of leaf expansion and vigorous growth, but decreased when the leaves were shed. In contrast, ABA/IAA and ABA/(IAA + ZR + GA) remained stable at the stages of leaf expansion and vigorous growth.

### 3.3. Photosynthetic Gas Exchange

**Figure 4:**
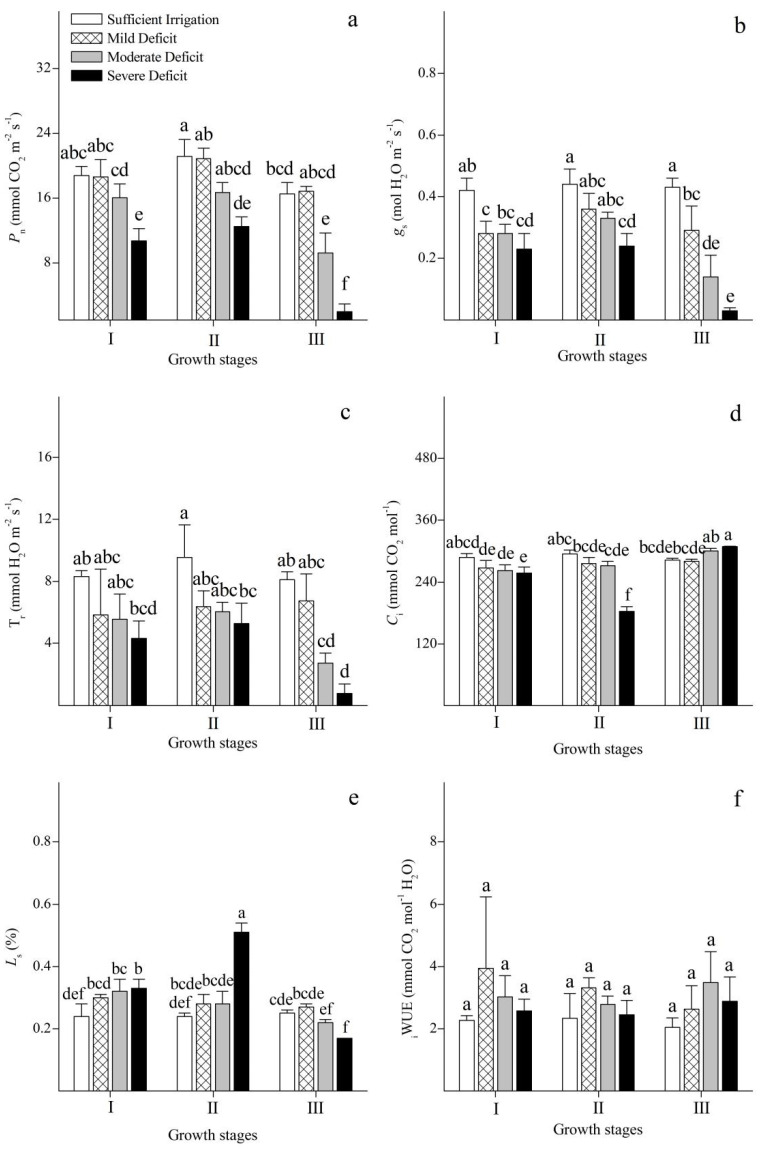
Changes in photosynthetic rate (*P*_n_) (**a**), stomatal conductance (*g*_s_) (**b**), transpiration rate (Tr) (**c**), intercellular CO_2_ concentration (*C*_i_) (**d**), (stomatal limitation value) *L*_s_ (**e**), and instantaneous water-use efficiency (_i_WUE) (**f**) of *Caragana korshinskii* Kom. under different irrigation strategies at different growth stages. I, II, and III stand for leaf expansion stage, vigorous growth stage, and leaf-shedding stage, respectively. Data points are mean ± SD of three replicates. Different letters indicate significant difference among the treatments.

The evolution of photosynthetic gas exchange in Korshinsk peashrub under different irrigation regimes was analyzed in this study (Figure 4a–f). *P*_n_ was higher at the stages of leaf expansion and vigorous growth, but lower when the leaves were shed. In terms of irrigation strategies, mild water deficit had no significant effect on the rate of photosynthesis at all stages of growth. However, the photosynthetic rate was significantly reduced under moderate and severe water deficits compared with that under sufficient irrigation.

Under sufficient irrigation, *g*_s_ and T_r_ could be maintained above 0.4 mol H_2_O_2_ m^−2^s^−1^ and 8 mmol H_2_O_2_ m^−2^s^−1^, respectively. However, with an increase in water deficit, *g*_s_ and T_r_ were gradually reduced. When the leaves were shed, *g*_s_ and T_r_ reached their lowest levels under severe deficit.

At the stages of leaf expansion and vigorous growth, *C*_i_ showed a trend towards reduction with the increment in levels of water deficit. However, when the leaves were shed, a gradual rise in *C*_i_ was observed as the degree of water deficit increased. *L*_s_ showed a different trend to that of *C*_i_, and instead rose with an increase in water deficit at the stages of leaf expansion and vigorous growth, but it was reduced with an increment in levels of water deficit when the leaves were shed. In addition, with an increment in levels of water deficit, the _i_WUE showed a trend of first increasing and then decreasing at each stage of growth.

### 3.4. Chlorophyll Fluorescence

**Figure 5:**
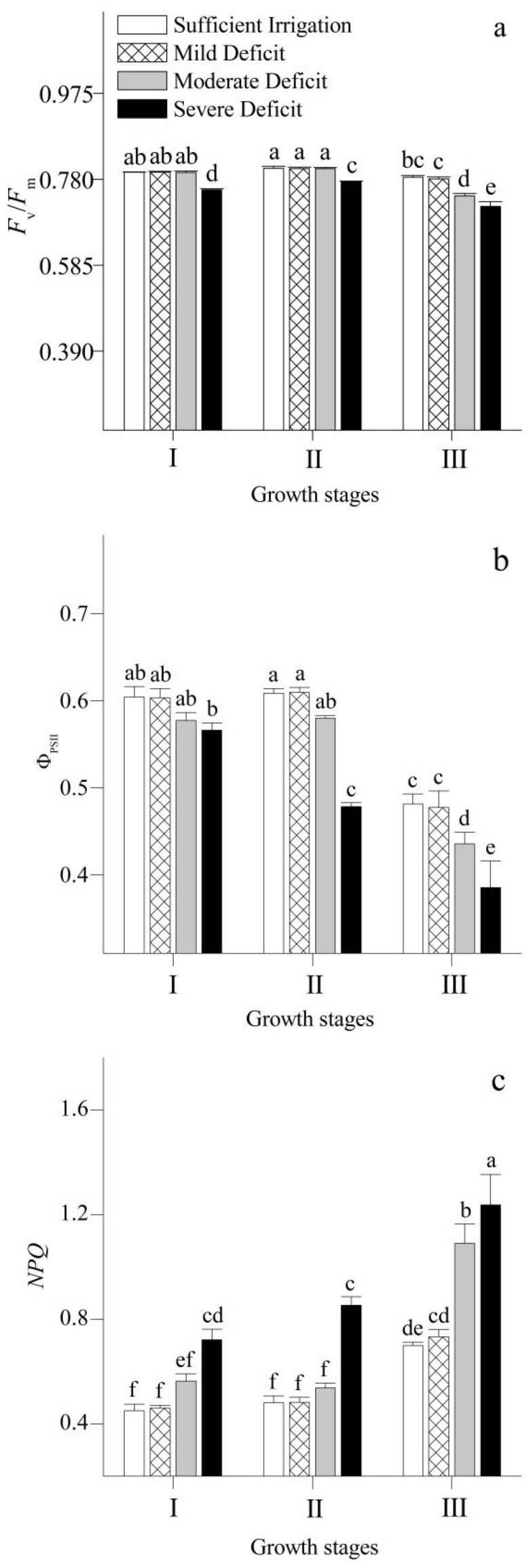
Changes in maximal efficiency of PSII (*F*_v_/*F*_m_) (**a**), actual efficiency of PSII (Φ_PSII_) (**b**), and non-photochemical quenching (*NPQ*) (**c**) of *Caragana korshinskii* Kom. under different irrigation strategies at different growth stages. I, II, and III stand for leaf expansion stage, vigorous growth stage, and leaf-shedding stage, respectively. Data points are mean ± SD of three replicates. Different letters indicate significant difference among the treatments.

The evolution of chlorophyll fluorescence in Korshinsk peashrub under different irrigation regimes is shown in Figure 5. *F*_v_/*F*_m_ and Ф_PSII_ were above 0.78 and 0.60 at the stages of leaf expansion and vigorous growth, but lower than 0.78 or 0.50 when the leaves were shed. At the stage of leaf expansion and vigorous growth, *F*_v_/*F*_m_ remained stable above 0.78 under mild and moderate water deficit conditions. However, when the leaves were shed, *F*_v_/*F*_m_ was significantly reduced to below 0.78 under moderate water deficit. During the entire growth stage, Ф_PSII_ in the leaves decreased under moderate and severe water deficits.

The amount of *NPQ* was higher when the leaves were shed than when observed at the stages of leaf expansion and vigorous growth. In terms of irrigation strategies, a mild water deficit had no significant effect on the amount of *NPQ* during the entire growth stage. Under moderate and severe water deficits, the amount of *NPQ* tended to increase.

### 3.5. Oxidative Damage and ROS-Scavenging Systems

**Figure 6:**
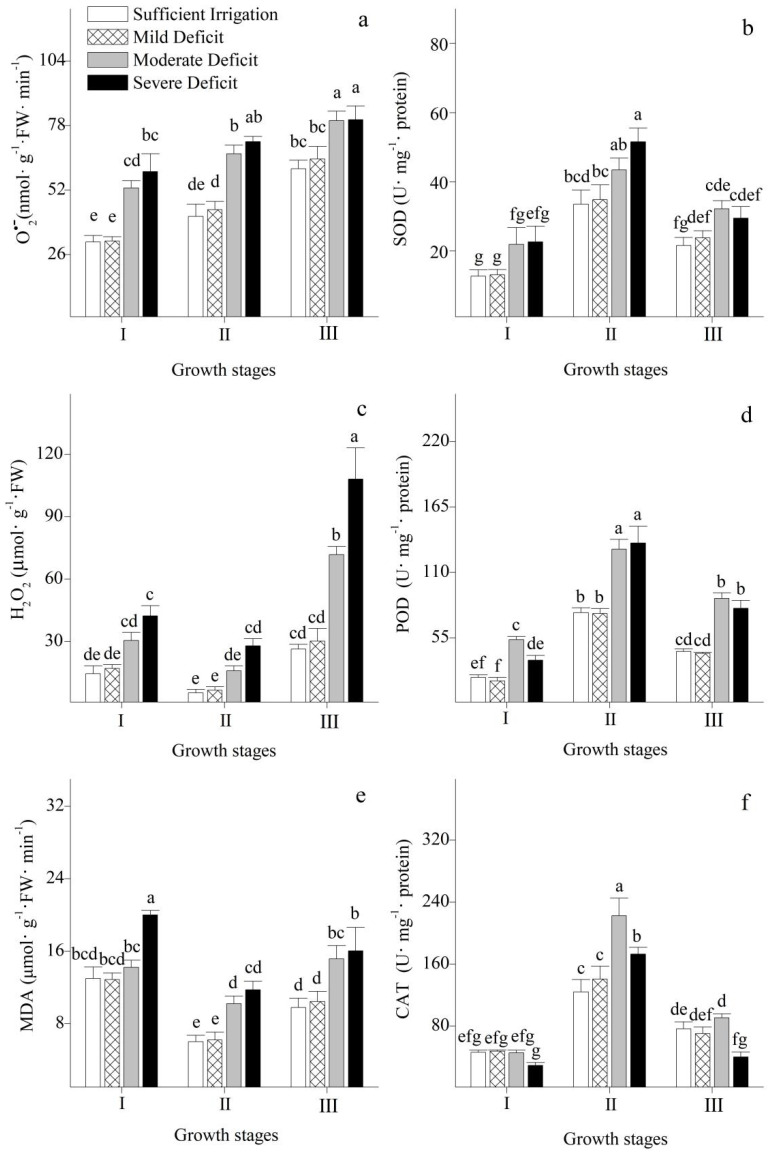
Changes in superoxide anion (O_2_^•−^) production rate (**a**), hydrogen peroxide (H_2_O_2_) concentration (**c**), malondialdehyde (MDA) concentration (**e**), superoxide dismutase (SOD) activity (**b**), peroxidase (POD) activity (**d**), and catalase (CAT) activity (**f**) of *Caragana korshinskii* Kom. under different irrigation strategies at different growth stages. I, II, and III stand for leaf expansion stage, vigorous growth stage, and leaf-shedding stage, respectively. Data points are mean ± SD of three replicates. Different letters indicate significant difference among the treatments.

The evolution of oxidative damage and ROS-scavenging systems in Korshinsk peashrub under different irrigation regimes was analyzed in this study (Figure 6a–f). The production rate of O_2_^•−^ and the concentration of H_2_O_2_ in the leaves when they were shed were higher than those observed at the stages of leaf expansion and vigorous growth. No significant changes in the production rate of O_2_^•−^ and the concentration of H_2_O_2_ in the leaves were observed under mild water deficit. However, under moderate and severe water deficits, the production rate of O_2_^•−^ and the concentration of H_2_O_2_ significantly increased. In addition, mild water deficit had no significant effect on the levels of MDA in the leaves, but the levels of MDA significantly increased under moderate-to-severe water deficit stress.

The activities of SOD, POD, and CAT in the leaves at the vigorous growth stage were higher than those observed when the leaves expanded or were shed. The activities of protective enzymes in the leaves of Korshinsk peashrub did not significantly change under mild water deficit. However, the activities of SOD and POD significantly increased under moderate and severe water deficits compared with those subject to sufficient irrigation. In addition, under severe water deficit, the activity of CAT in the leaves at stages of leaf expansion and shedding was significantly lower than that during sufficient irrigation.

## 4. Discussion

Different irrigation strategies have different effects on the growth and development of plants. In our study, mild and moderate water deficits had no significant effect on the biomass of Korshinskii peashrub, but severe water deficit significantly reduced the fresh and dry weights in its branches and leaves (Figure 1a,b). This appears to be related to the inhibition of branch and leaf growth caused by changes in the physiological traits under severe water deficit.

In this study, the concentrations of IAA, ZR, and GA in the leaves at the stages of leaf expansion and vigorous growth were maintained at a relatively high level. This promotes cell division and elongation, which facilitates the growth of Korshinskii peashrub. There was a high concentration of growth-inhibiting hormone in the leaves during their final stage, which leads to leaf senescence and shedding. The concentration of ABA in the leaves increased as the levels of water deficit gradually increased. This phenomenon could be owing to a decrease in the breakdown and/or an increment in the synthesis of ABA in the roots that is induced by water deficit [23], and the transport of ABA from the roots to shoots increased [24]. This would result in the closure of stomata and thereby reduce the consumption of water by the plant. In contrast, there was an acceleration in the increase in the breakdown and/or a decrease in the synthesis of ZR and GA with an increased water deficit [25]. Thus, their concentrations gradually decreased in the leaf tissues. As a result, plant cell division and leaf elongation are delayed and the tolerance of the plant to stress is enhanced. In addition, at the stages of leaf expansion and vigorous growth, the concentration of IAA in the leaves gradually increased with an increment in levels of water deficit, which could be explained by the fact that tryptophan, a precursor of IAA, increases under water deficit [26,27]. However, when the leaves were shed, the concentration of IAA was gradually reduced as the water deficit level increased. This phenomenon could be owing to the involvement of IAA in the physiological progress of adaptation to water deficit and its participation in phase-specific functions [27]. These results indicated that under moderate and severe water deficits, the growth of Korshinsk peashrub could be maintained at the stages of leaf expansion and vigorous growth, but was restricted at the leaf-shedding stage.

Many endogenous hormones are involved in the regulation of leaf physiological processes. Indeed, leaf physiological metabolism is coordinated by the interaction of various hormones [28]. To more effectively explore the physiological regulation of endogenous hormones on physiological processes, the interaction and balance mechanism of endogenous hormones in Korshinsk peashrub leaves under different irrigation strategies were analyzed. At the stages of leaf expansion and vigorous growth, the ratios of growth-inhibiting to growth-promoting hormones were relatively low, and ABA/IAA and ABA/(IAA + ZR + GA) remained stable under water deficit; this is conducive to mesophyll cell division and elongation [29]. However, at the leaf-shedding stage, the ratios of leaf growth-inhibiting to growth-promoting hormones were maintained at a high level, and they gradually increased with an increment in levels of water deficit. This results in the inhibition of mesophyll cell division and elongation, and accelerates leaf senescence and shedding [30].

Photosynthesis is the most important biochemical process by which plants convert light energy into stable chemical energy. Many studies have reported that water deficit either directly or indirectly restricts photosynthesis [9,31]. During the initial stage of water deficit, ABA in the roots were immediately synthesized, and could promote stomatal closure and, thus, inhibit the uptake of CO_2_ [32]. When the concentration of intercellular CO_2_ cannot satisfy the requirements for photosynthetic carbon assimilation, photosynthesis is restricted by stomatal factors [2]. With progressive water deficit, non-stomatal factors, such as mesophyll resistance and PSII damage, also occur and restrict the assimilation of photosynthetic carbon in coordination with stomatal factors [33]. Stomatal contraction reduces the levels of intercellular CO_2_, but non-stomatal limitation of photosynthesis could result in the accumulation of CO_2_ in the intercellular space [34]. Therefore, the most prominent factor that restricts photosynthesis under water deficit could be confirmed by the changes in *C*_i_ and *L*_s_ [7]. The results of our studies suggested that water deficit decreases stomatal conductance and restricts the entry of CO_2_ into the intercellular space. At the stages of leaf expansion and vigorous growth, the carbon assimilation of Korshinsk peashrub was maintained at a high level, and there was a large demand for CO_2_. Water deficit led to a decrease in *C*_i_ and an increase in *L*_s_ in the leaves, which suggests that stomatal limitation is the primary factor that inhibits the assimilation of photosynthetic carbon. At the leaf-shedding stage, stomatal closure was induced by water deficit, and lower concentrations of CO_2_ entered the intercellular space. However, with the restriction of photosynthetic apparatus senescence and loss of function, there was a relatively lower demand of CO_2_ for carbon assimilation. This resulted in an increase in *C*_i_ and a decrease in *L*_s_, which indicated that the non-stomatal factor became the major factor in limiting photosynthesis.

Chlorophyll fluorescence, one of the most practical techniques used to understand the physiological mechanisms of photosynthesis, can provide detailed information about the state of PSII under different conditions [35]. Our study analyzed changes in the chlorophyll fluorescence of Korshinsk peashrub at different growth stages under water deficit. At the stages of leaf expansion and vigorous growth, *F*_v_/*F*_m_ and Ф_PSII_ remained at a relatively high level. Under moderate water deficit, Ф_PSII_ was downregulated with an increase in *NPQ* to avoid the possible physiological damage caused by energy excess excitation to the PSII reaction center. Thus, excessive amounts of excitation energy were dissipated, and *F*_v_/*F*_m_ was maintained [2]. With progressive water deficit, a continuous decrease in Ф_PSII_ coincided with a continuous increase in *NPQ*, but the photo-protective mechanism under severe water deficit was not enough to avoid photo-damage. A significant reduction in *F*_v_/*F*_m_ occurred, and non-stomatal factors were involved in the inhibition of photosynthesis. When the leaves were shed, *F*_v_/*F*_m_ and Ф_PSII_ were lower than those at the stages of leaf expansion and vigorous growth. Under mild water deficit, *F*_v_/*F*_m_ could be maintained by downregulating Ф_PSII_ and increasing *NPQ* to dissipate excess excitation energy. However, *F*_v_/*F*_m_ significantly decreased under moderate water deficit.

Under water deficit, stomatal closure induced an imbalance between PSII activity and the electron requirement of photosynthesis [36] and, therefore, excessive electrons were transferred to molecular oxygen. Molecular oxygen cannot simultaneously accept four electrons to produce H_2_O; it can only accept one electron at a time and is gradually reduced to an intermediate product of ROS [37]. Molecular oxygen initially received an electron to generate O_2_^•−^ [38], which led to a rapid increase in the production rate of O_2_^•−^ in the leaves under moderate and severe water deficits. O_2_^•−^ can both oxidize and reduce. It not only inhibits photosynthetic enzyme activities but also directly attacks proteins and nucleic acids, which causes damage to the photosynthetic apparatus [14,39]. In addition, O_2_^•−^ can help to initiate the expression of genes that encode SOD as intermediate messengers to upregulate its activity [40] and be rapidly disproportioned or spontaneously converted into H_2_O_2_ [39]. Therefore, the activity of SOD significantly increased with an increase in the production rate of O_2_^•−^. In addition, the concentrations of H_2_O_2_ also tended to increase under moderate and severe water deficits. To avoid possible physiological damage caused by the accumulation of H_2_O_2_, the activities of antioxidative enzymes, such as POD and CAT, significantly increased under moderate water deficit, and were conducive to scavenging for excess H_2_O_2_ and maintaining the oxidation–reduction balance in plant cells. However, severe water deficit reduced the activity of CAT. When the protective enzyme systems were insufficient in eliminating excessive H_2_O_2_, the oxidative damage induced by water deficit was observed in the leaves. In our study, the concentration of MDA in the leaves reached its highest level under severe water deficit, which indicated that severe water deficit caused the most serious over-oxidative damage to cellular membrane lipids.

In conclusion, with an increase in water deficit, the concentration of ABA in the leaves dramatically increased, while growth-promoting hormones, such as ZR and GA, gradually decreased. At the stages of leaf expansion and vigorous growth, Φ_PSII_ was downregulated with an increase in *NPQ*; photo-damage was avoided, and *F*_v_/*F*_m_ was maintained under moderate water deficit. However, with progressive water stress, the photo-protective mechanism was not enough to avoid photo-damage; *F*_v_/*F*_m_ decreased, and the non-stomatal inhibition of photosynthesis occurred. At the leaf-shedding stage, non-stomatal factors became the major factor in limiting photosynthesis under moderate and severe water deficits. In addition, the production rate of O_2_^•−^ and concentration of H_2_O_2_ rapidly increased with an increase in water deficit, and SOD and POD significantly increased to eliminate excess ROS. Ultimately, the over-oxidative damage was still significantly increased under severe water deficit.

## Data Availability

Not applicable.
